# Spotting missing or wanted people: racial biases in prospective person memory

**DOI:** 10.1186/s41235-024-00597-z

**Published:** 2024-10-08

**Authors:** Megan H. Papesh, Daniella K. Cash, Juan D. Guevara Pinto, Sofia V. Lomba

**Affiliations:** 1 Department of Psychology, University of Massachusetts Lowell, Lowell, MA USA; 2 Sam Houston State University, Huntsville, TX USA; 3 Rollins College, Winter Park, FL USA

## Abstract

Searching for missing or wanted people is a crucial task in our society. Previous work on prospective person memory (PPM) has demonstrated that performance on this type of search task is worse relative to standard prospective memory tasks. Importantly, this process may be further affected by the race of the missing person, yet this has never been tested in laboratory settings. To test the effects of race on PPM, a convenience sample consisting primarily of self-identified Caucasian participants was asked to search for either a Caucasian or an Indian target person while judging the orientation of different Caucasian and Indian faces. Although the tasks were otherwise identical, 89% of Caucasian PPM targets were found while only 53% of Indian targets were found. Furthermore, relative to a control group with no PPM requirements, participants were slower and more error-prone when judging Indian faces relative to White faces, particularly if they were searching for an Indian face. We interpret these results as revealing other-race effects in prospective person memory, highlighting race as a critical factor for finding missing people. Importantly, this also emphasizes the need for real-world search efforts to factor in difficulty differences when people monitor for missing/wanted people from their own or different racial backgrounds. For example, media coverage of missing persons cases could perhaps be distributed more equitably by considering whether the missing person is from a racial minority in that region.

## Introduction

According to the National Crime Information Center’s (NCIC’s) Missing Person and Unidentified Person statistics, there were over 97,000 active missing persons reports in the United States at the end of the 2022 calendar year. As noted by the Black and Missing Foundation, non-White minorities make up nearly 40% of all missing persons cases. When someone goes missing, friends, family, and the authorities will often solicit help from the public by releasing photographs or physical descriptions of the missing person. Depending on where one lives, these are often seen in AMBER alerts, Silver alerts, missing person posters/billboards, and social media campaigns, among others. When a person of color goes missing in the United States, the audience targeted by these varied strategies may be primarily comprised of other-ethnicity individuals. Moreover, search campaigns may not be as widely distributed for non-White minorities. Research has shown that the cases of some minority groups (e.g., Native American women) receive little-to-no coverage in popular media (Parsloe & Campbell, 2021). The present research explores the impact of searching for missing/wanted people from different racial/ethnic groups.

In scientific terms, searching for missing persons relies on searchers effectively utilizing two types of memory, prospective and retrospective memory (Lampinen et al., 2009a; McDaniel & Einstein, 2000). Although there are multiple types of prospective memory (PM), prospective *person* memory is a sub-class of event-based PM. In event-based PM, individuals must remember to fulfill an intention when a specific event occurs (Einstein & McDaniel, 1990). For example, remembering to fill a prescription (intention) when passing the drugstore (event). Laboratory studies of event-based PM typically require participants to engage in an ongoing task (e.g., lexical decisions) while remaining vigilant for one or more PM targets (e.g., specific words or classes of words). When a target is encountered, participants are often asked to abandon the primary task and issue a different response. Research using this paradigm yields several measurable behaviors and consistent effects of different manipulations on those behaviors. For example, ongoing task response time is often slower and responses are less accurate when participants monitor for multiple or vague prospective memory targets (e.g., Cohen et al., 2008; Hicks et al., 2005; Lourenço et al., 2015) and their incidental memory for non-target items is higher when the PM targets are more difficult to monitor for (e.g., Guevara Pinto et al., 2021).

Prospective person memory studies typically use similar protocols to event-based PM studies. For example, after forming an intention to look for a specific person, participants might classify faces into distinct groups or “teams” while trying to remember to abandon the classification task when they spot the missing person (e.g., Lampinen et al., 2012a; Sweeney & Lampinen, 2012). Participants typically do not perform as well on prospective person memory tasks as they do on standard event-based PM tasks. This deficit remains when participants view age-progressed photographs (Lampinen et al., 2012b) or even videos of the missing person (Lampinen & Moore, 2016a). Even when the prospective person memory tasks are made more realistic (e.g., by having participants encounter a “wanted” person while engaged in other activities on campus), detection rates remain low (Lampinen et al., 2009b, 2015).

Why is prospective person memory seemingly more challenging than standard event-based prospective memory? According to Lampinen and Moore (2016b), successfully spotting a missing or wanted person requires several processes. These include (1) Encountering the alert, (2) Attending to the alert, (3) Encountering the missing person, (4) Attending to the missing person, (5) Remembering the missing person’s face, from where it is known, and the intention to contact authorities, and (6) Contacting the authorities. Although there is room for error in any of those steps, arguably the most cognitively demanding stage is step 5: Remembering the face, its source, and that the face is associated with an intention to notify authorities. Although increasing one’s expectations that they will encounter the face improves the likelihood that one will attend to the missing person (step 4; Moore et al., 2016), to date, only providing multiple photographs of the missing person seems to increase participants’ ability to remember the missing person (Sweeney & Lampinen, 2012).

Remembering the missing person relies on retrospective memory abilities. Participants must successfully encode the face and avoid confusing it with other, similar faces. This task is more challenging than many may realize; although familiar face memory is often exceptional and robust to changes in age and physical appearance (Laurence et al., 2021), unfamiliar face memory is often exceptionally poor (Megreya & Burton, 2006). Unfamiliar face memory is further challenged by well-established other-race effects: People tend to have worse memory for faces from ethnicities other than their own, particularly when the other race is a minority group in that region (Lee & Penrod, 2022). Although other-race effects do not always emerge in laboratory studies (e.g., Gier & Kreiner, 2023, did not observe an own-race bias in retrospective memory for an older Black couple appearing in a Silver Alert), they remain fairly robust even in multicultural settings (e.g., Wong et al., 2020). Explanations for other-race effects are numerous and include perceptual expertise (Tanaka et al., 2013) and social-cognitive (Hugenberg et al., 2010) accounts. Regardless of their source, other-race effects often manifest in memory studies as greater confusability between other-race faces, producing higher false alarm rates on old/new memory tests (Meissner & Brigham, 2001). In the context of a PPM task, these false alarms could potentially produce *better* performance for other-race face detection, relative to own-race face detection.

 Given the deficits observed in retrospective memory for other-race faces, we sought to determine the impact of ethnicity-specifying information on prospective person memory. Because nearly 40% of missing persons are non-White, other-race biases in prospective person memory could have a sizeable effect on the resolution of missing person’s cases.^1^ To that end, we investigated how search for other-race faces affects (a) PM performance, (b) ongoing task performance, and (c) incidental learning of other faces. We predicted differences in Indian and Caucasian PM detection rates. If the differences result from greater confusability between other-race faces, participants should have a higher detection rate for Indian PM targets, driven by an inflated false alarm rate. Additionally, because research shows that other-race faces are more challenging to learn and remember, we also hypothesized that participants would show slower (and more error-prone) ongoing task performance.

## Method

### Participants

A convenience sample of 77 students and staff members (29.87 years old, *SD* = 13.01) were recruited from a small liberal arts college in the southern United States and compensated with a $10 Amazon gift card. We aimed to recruit at least 23 participants per condition, as suggested by Wong et al. (2020). A post hoc power analysis confirms sufficient power to detect medium effects. Most participants self-reported as White/Caucasian American (*n* = 60), but other ethnicities were also represented (10 Asian/Asian American, 6 Black/African American, 1 Native American/Alaska Native). Participants were randomly assigned to one of two PM Conditions, which determined the ethnicity of the missing person (Indian or Caucasian), or to a control condition with no PM intention.

### Materials

We created 128 unique identities using Generated.Photos (Generated Media, Inc., 2019)^2^ with half representing a White male and the other half representing an Indian male. To isolate the effect of ethnicity, we ensured that the age, emotional expression, and hair length were the same across both Caucasian and Indian ethnicities. Faces for both the Indian and Caucasian ethnicities were created using the randomize function until the appropriate number of identities had been created. Each picture included the full face and were cropped at the shoulders to avoid any additional retrieval cues via clothing. Two images were created for each identity: One in which the face was oriented forward toward the observer, and one in which the face was oriented either leftward or rightward (64 left, 64 right; see Appendix, for examples). Out of the 128 distinct identities, 8 were pseudo-randomly selected to be used as practice stimuli, 24 were selected to serve as potential PM targets, and the remaining 96 were used as nontarget stimuli. To avoid testing picture memory, the left- and right-facing photographs were used in the PM phase and the front-facing photographs were used in the recognition phase. Experiment materials for this study can be accessed at: https://osf.io/gtvfd/?view_only=820d0b5fb3774ba9a9174ef857c03230

### Procedure

All materials and procedures were approved by the IRB at the data collection site. After providing written informed consent, participants completed the experiment individually on a laptop computer in quiet laboratory room. Participants provided demographic details then completed 8 practice trials in which their task was to quickly determine whether a centrally presented face was oriented to the left (press “F”) or right (press “J”) of the screen. Feedback was presented for 500 ms following each trial.

After the practice trials, participants were randomly assigned to one of three between-groups conditions to determine their PM task. Two groups were given a PM intention (monitoring for either a Caucasian or Indian “missing/wanted person”) and the Control group had no PM intention. Participants in the PM conditions were instructed that they would continue to make orientation decisions, but now they would also monitor for a specific face (PM target) and provide a different response for it (press “B”) instead of a left/right judgment. Based on condition, PM target identities were randomly selected for each participant from a pool of 12 Caucasian or 12 Indian faces. Participants studied the PM target and indicated when they were ready to continue. After, participants repeated the task instructions verbally to the researcher to ensure they understood the task and then proceeded to the main experimental phase. Participants in the Control condition completed the same face orientation task as they had earlier in the practice trials. Consistent with previous PM research (e.g., Marsh et al., 2005, 2006), all participants waited for three minutes after receiving their condition-specific instructions before beginning the experimental phase.

Throughout the experimental phase, each nontarget face was repeated three times, resulting in 144 total trials with race and left/right orientation selected randomly but equally. For the PM intention conditions, eight additional trials were included in which the PM target was presented (yielding 152 trials total). These eight PM targets were randomly presented throughout the experiment, with the limitations that two PM targets could not be presented consecutive after each other.

Following the main experimental phase, participants completed a surprise old/new recognition test for the *nontarget* faces they encountered throughout the ongoing task. The recognition test presented 96 front-facing faces sequentially (half old identities and half new, with equal representation of Caucasian and Indian identities) which participants judged as old or new using the “F” and “J” keys (response mapping was counterbalanced across participants). No feedback was provided. After the completion of the recognition test, participants were thanked and compensated.

## Results

Prior to analysis, we examined the data for response time (RT) outliers, which we defined as ongoing task trials with RTs shorter than 200 ms or greater than 2.5 standard deviations above the individual participant mean (separately for the control and PM groups). This resulted in 3.8% and 4.2% of trials being dropped from the analysis for the control and PM conditions, respectively. Type I error rate was set to 0.05 and maintained in multiple comparisons with Bonferroni corrections. All analyses were conducted using JASP (JASP Team, 2023). Data analysis files for this study can be accessed at^3^: https://osf.io/gtvfd/?view_only=820d0b5fb3774ba9a9174ef857c03230

### PM performance

To determine whether participants’ PM performance was affected by the race of the missing/wanted person, we compared PM hit rates for participants in the PM intention conditions in an independent samples *t* test. Detection rates were higher for participants monitoring for a Caucasian person (*M* = 0.89, SE = 0.03) than those monitoring for an Indian person (*M* = 0.53, SE = 0.08), *t*(50) = −4.47, *p* < 0.001, Cohen’s *d* = −1.24. Although detection accuracy differed, the time necessary for participants to successfully detect a missing/wanted person was unaffected by race, *t*(45) = 0.63, *p* = 0.53, Cohen’s *d* = 0.19. Furthermore, only four participants failed to successfully detect any PM targets (all from the Indian PM condition), and the PM hits analyses were unchanged when these participants were excluded, *t*(45) = −3.39, *p* < 0.001, Cohen’s *d* = 0.99.

### Ongoing task performance

**Fig. 1:**
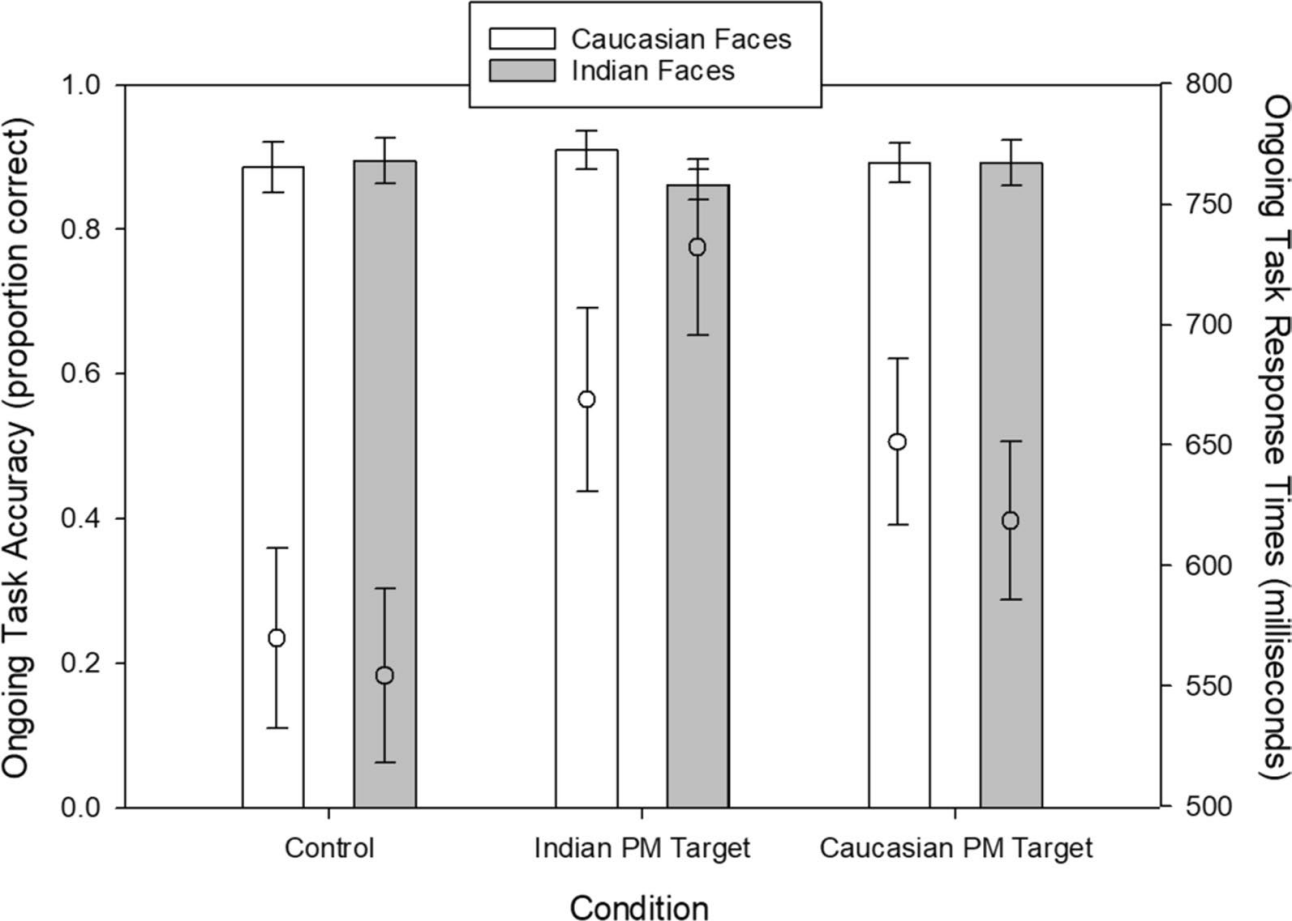
Ongoing task performance across conditions and trial type. Bar graphs represent ongoing task accuracy (y-axis on left side) and embedded dot represents ongoing task response times (y-axis on right side). White colored bars and dot reflect trials where a Caucasian face was presented. Gray colored bars and circles reflect trials where an Indian face was presented. All error bars reflect ± 1 SEM

In the PM literature, ongoing task performance (accuracy and RT) is typically detrimentally affected by the difficulty of the PM monitoring task. To determine whether the race of the missing/wanted person similarly affects ongoing task performance, we analyzed participants’ face orientation accuracy and RT in separate 2 (Nontarget Face Race: Caucasian, Indian) × 3 (Condition: Caucasian PM Target, Indian PM Target, Control) mixed model ANOVAs, both with Condition as the between-subjects variable. Accuracy analyses revealed a reliable interaction, *F*(2, 74) = 7.21, *p* = 0.001, *n*^*2*^_*p*_ = 0.16, and no other effects. As shown in the bar graph in Fig. 1, participants monitoring for Indian faces had lower ongoing task accuracy when judging Indian faces, relative to when they judged Caucasian faces, *t* = 4.22, *p* = 0.001, Cohen’s d = 0.32. No other post hoc tests were reliable.

Analysis of ongoing task RTs revealed a main effect of Condition, *F*(2, 74) = 3.73, *p* = 0.003, *n*^*2*^_*p*_ = 0.09, and a reliable interaction, *F*(2, 74) = 39.77, *p* < 0.001, *n*^*2*^_*p*_ = 0.52. As shown in the dot graph in Fig. 1, participants in the Control condition were generally fastest and did not show any differences based on the race of the faces. By contrast, participants in the PM conditions made slower decisions overall, particularly when the nontarget face being judged was of the same race as the PM target face, both *p*s < 0.05. Importantly, post hoc tests revealed this difference to be larger for the Indian PM Target condition (*p* < 0.001) than for the Caucasian PM Target condition (*p* = 0.002). Consistent with the analyses of ongoing task accuracy, these findings suggest that a larger attentional “cost” is demanded when searching for an Indian face relative to a Caucasian face.

### Memory performance

**Fig. 2:**
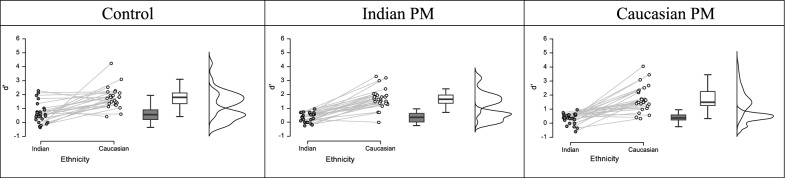
Raincloud plots generated in JASP showing individual and distributional data for d’ for the Control condition (left column), Indian PM Target (middle column) and Caucasian PM Target (right column)

**Fig. 3:**
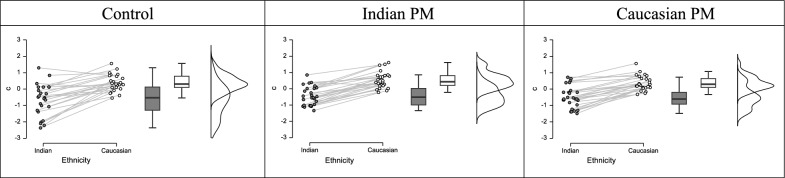
Raincloud plots generated in JASP showing individual and distributional data and c for the Control condition (left column), Indian PM Target (middle column) and Caucasian PM Target (right column)

To determine whether the difficulty of monitoring for an other-race face produced incidental recognition benefits similar to those that emerge when PM target difficulty is manipulated (e.g., Guevara Pinto et al., 2021), we used participants’ hit and false alarm rates in the surprise memory test to calculate the signal detection indexes *d’* (sensitivity; Fig. 2) and *c* (bias; Fig. 3). We analyzed both measures in separate 2 (Nontarget Face Race: Caucasian, Indian) × 3 (Condition: Caucasian PM Target, Indian PM Target, Control) mixed model ANOVAs. Both analyses only revealed reliable effects of Nontarget Face Race, *d’*: *F*(1, 74) = 141.76, *p* < 0.001, *n*^*2*^_*p*_ = 0.66; *c*: *F*(2, 74) = 208.53, *p* < 0.001, *n*^*2*^_*p*_ = 0.74 As shown in the raincloud plots in Figs. 2 and 3 (Allen et al., 2019), participants in all conditions had higher sensitivity for Caucasian faces, relative to Indian, and a more liberally skewed bias for Indian faces, relative to Caucasian.

## Discussion

Non-White minorities make up nearly 40% of all missing persons cases (NCIC, n.d.), which means that the AMBER alerts, Silver alerts, billboards, and other media used to help locate many missing people are often directed toward individuals from other racial/ethnic backgrounds. Search for missing/wanted people involves both retrospective and prospective components of memory (Lampinen et al., 2009a): Individuals must learn the face of the missing person while also remembering to monitor for them and alert the authorities should they encounter them. Although other-race deficits in long-term memory for faces have long been established (e.g., Lee & Penrod, 2022; Meissner & Brigham, 2001), the present study sought to determine whether these deficits also exist in prospective person memory. We conducted a simplified laboratory version of what ideally happens when one is notified of a missing or wanted person in their area: Predominantly Caucasian participants learned the face of the missing person and then carried out an unrelated ongoing task under instructions to abandon that task when they encounter the missing person’s face. At the end of the ongoing task, we tested participants’ memory for the people they encountered. Participants’ long-term and prospective person memories showed evidence of racial biases: They were less accurate detecting Indian missing people, and they had poorer memory for the Indian faces encountered throughout the ongoing task. During the ongoing task, performance was most disrupted when participants monitored for an Indian face, suggesting that doing so imposed greater cognitive challenge than monitoring for a Caucasian face.

Prospective person memory shares some qualities of event-based prospective memory (PM). For example, event-based PM research consistently shows that participants are slower and less accurate when they monitor for challenging PM targets (e.g., vague cues, multiple cues; Cohen et al., 2008; Hicks et al., 2005; Lourenço et al., 2015). In the present study, the challenge of the PM targets was enacted via a race manipulation: Participants were less accurate, although not necessarily slower, spotting other-race faces. This may reflect some of the unique challenges that distinguish prospective person memory from other forms of PM: Unfamiliar faces are challenging to recognize, particularly when they are from another racial background than the observer. Indeed, research on unfamiliar face memory often shows that memory for other-race faces only improves following differentiation instructions (e.g., Hugenberg et al., 2007). Although participants needed to differentiate the identity of the missing person from the non-target faces encountered in the task, their PM performance shows that they experienced limited success in this endeavor, suggesting that they could not necessarily differentiate the PM identity from the others. Whether these failures emerge from deficits in learning or deficits in task engagement remains an open question for future research.

While the results of the present study may provide discouraging information about the utility of missing persons alerts for helping to locate non-White people, the prospective person memory literature may offer some suggestions for improving this important task. For example, Lampinen and Moore (2016a) suggest that wide dissemination of missing person alerts and tactics to increase the likelihood that people *attend* to those alerts are key factors for resolving missing persons cases. Our results highlight the need for campaign efforts to be distributed in equity fashion (rather than equality), as there are measurable performance costs associated with monitoring for other-race faces. The photographs used in the alerts are also important. When the photographs are better representations of the missing person (e.g., cleanliness, age), people are better able to spot them (Gier et al., 2012; Lampinen et al., 2012a). Sweeney and Lampinen (2012) found that missing persons alerts are more effective when they contain multiple images of the missing person, which seem to better allow observers to extract consistent identity-specifying information.

The present results are based on a limited sample from a college in the southern United States and thus should be replicated with a wider and more diverse sample. For example, research showing that increased contact with other races can reduce or eliminate the own-race bias (e.g., Fioravanti-Bastos et al., 2014; Walker & Hewstone, 2006; Wright et al., 2003), which may make it more challenging to observe other-race effects in labs in demographically diverse locations. Although this is generally a good thing, it may discourage researchers from continuing to investigate the ways that social factors may impair cognitive processes. Our results add to the growing literature showing that other-race effects still emerge in face perception/recognition tasks regardless of local population demographics (e.g., Wong et al., 2020), and that these effects may scale up to impact the successful resolution of missing persons cases.

According to the 2022 AMBER Alert Report compiled by the National Center for Missing & Exploited Children, 181 AMBER alerts were issued in the 2022 calendar year. Of those, 16 were successfully resolved and linked to someone seeing and acting upon the alert. Although any one person’s likelihood of encountering a missing person is low, understanding the factors that affect the prospective person memory processes involved is critical when designing recommendations to increase the number of resolved cases. In the present research, we found that individuals monitoring for other-race faces were less successful at spotting missing people than individuals monitoring for Caucasian faces. Prior research has noted that identifying a missing person is a complex, multi-stage process (e.g., Lampinen & Moore, 2016a, 2016b), one in which failure at any stage could result in failing to recover the missing individual. The present research highlights an additional (and critical) barrier to the success of this process and suggests the need for additional research to overcome this obstacle.

## Figures and Tables

**Fig. 4:**
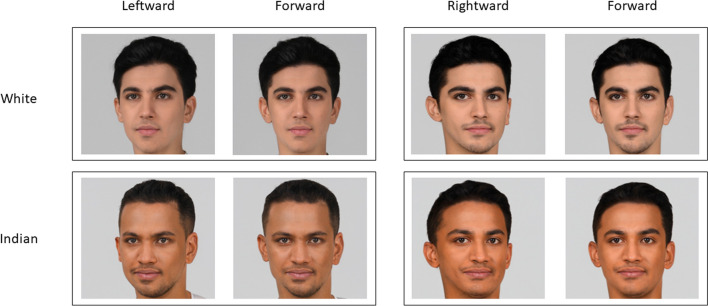
Examples of White and Indian faces used in the experiment. Individual identities are represented within each black box

## Data Availability

All data and materials are available on OSF (https://osf.io/gtvfd/?view_only=820d0b5fb3774ba9a9174ef857c03230).

## Appendix

See Fig. 4.

## Footnotes

1 Note that we are only focused on the cognitive mechanisms of search for own- and other-race persons. Social factors (e.g., motivation, media exposure) are also likely to influence this process, but are not examined in the current study.

2 See https://deepgram.com/ai-apps/generated-photos

3 All analyses were conducted on both the full dataset and the data restricted to only Caucasian participants. Across all measures, the effects and patterns were similar, so we report only the full analyses in text. Restricted analyses can be found on the OSF page.
